# Supplementary figures and images for: Near-term forecasting of companion animal tick paralysis incidence: An iterative ensemble model
Source: PLoS Comput Biol. 2022 Feb 16;18(2):e1009874. doi: 10.1371/journal.pcbi.1009874 (PMC8887734; doi:10.1371/journal.pcbi.1009874)

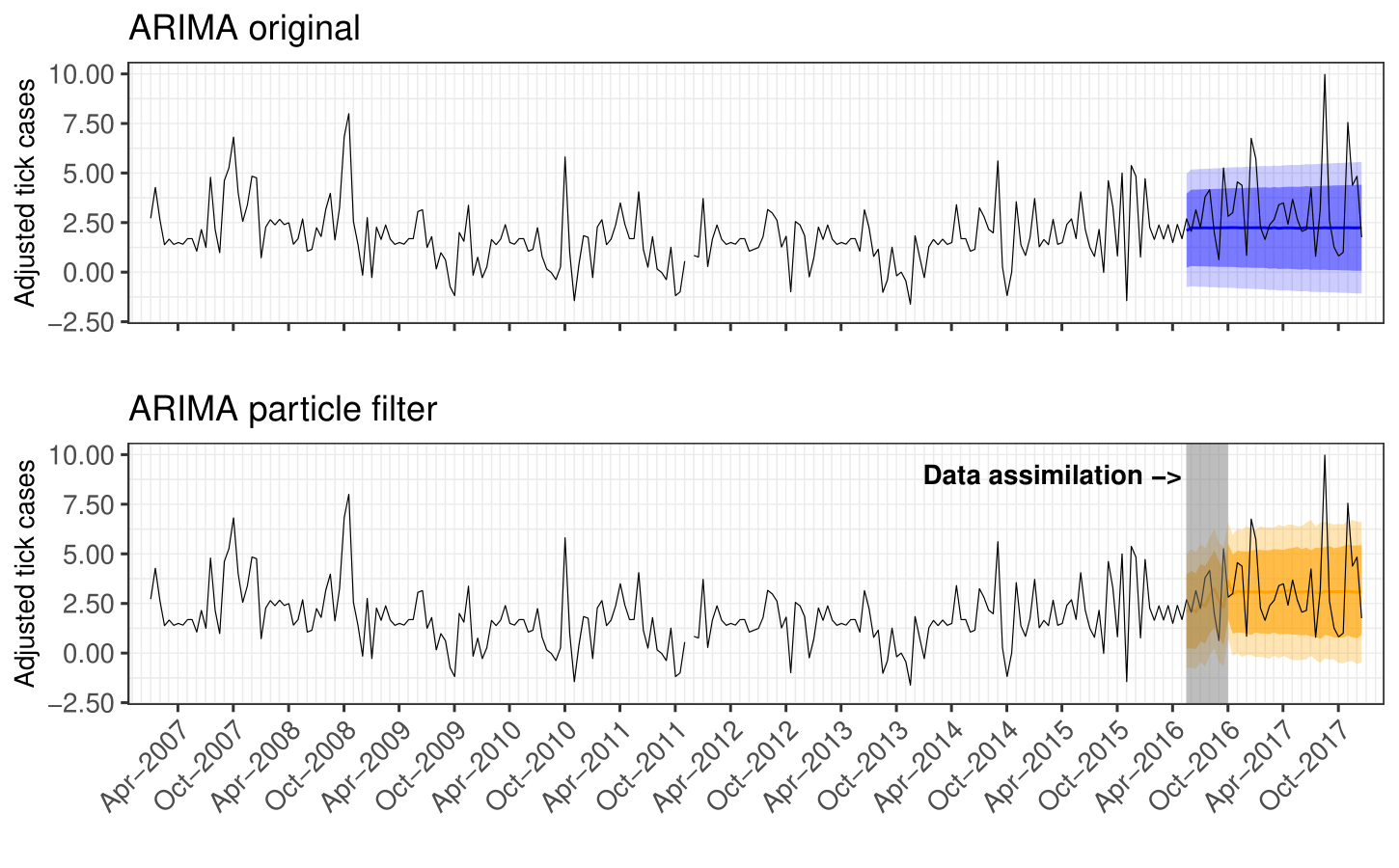

Supplement: S3 Fig — Forecasts of seasonally adjusted paralysis tick admissions (truth shown as the black line) generated by the original ARIMAseasadj (blue shading) and the ARIMAseasadj following particle filtering assimilation of the first six months of observations in the out-of-sample validation set (orange shading). For both forecasts, dark coloured shading shows 80% and light shading shows 95% prediction intervals. (TIFF) [file pcbi.1009874.s003.tiff]

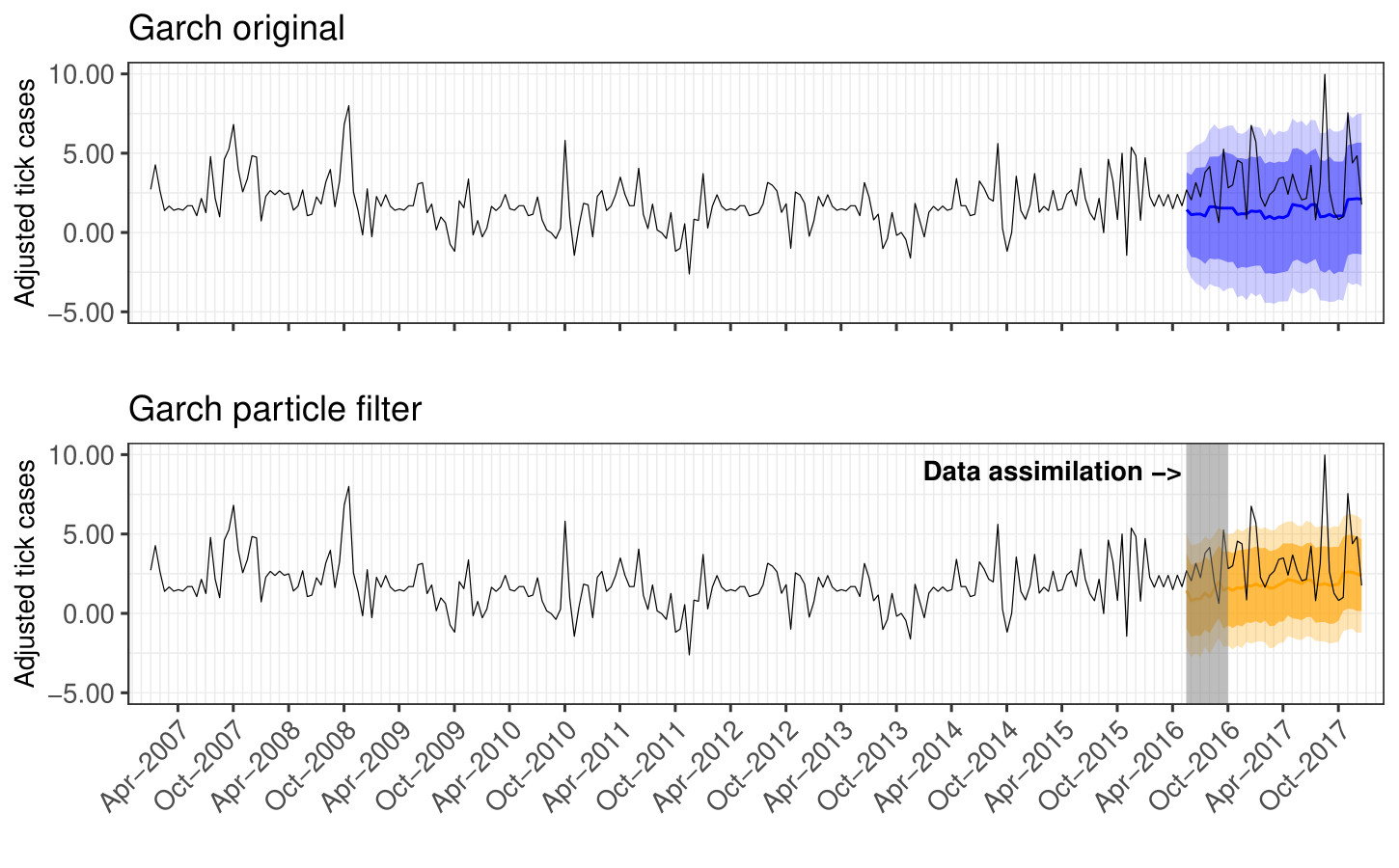

Supplement: S4 Fig — Forecasts of seasonally adjusted paralysis tick admissions (the true observations are shown as the black line) generated by the original GARCHseasadj (blue shading) and the GARCHseasadj following particle filtering assimilation of the first six months of observations in the out-of-sample validation set (orange shading). For both forecasts, dark coloured shading shows 80% and light shading shows 95% prediction intervals. (TIFF) [file pcbi.1009874.s004.tiff]

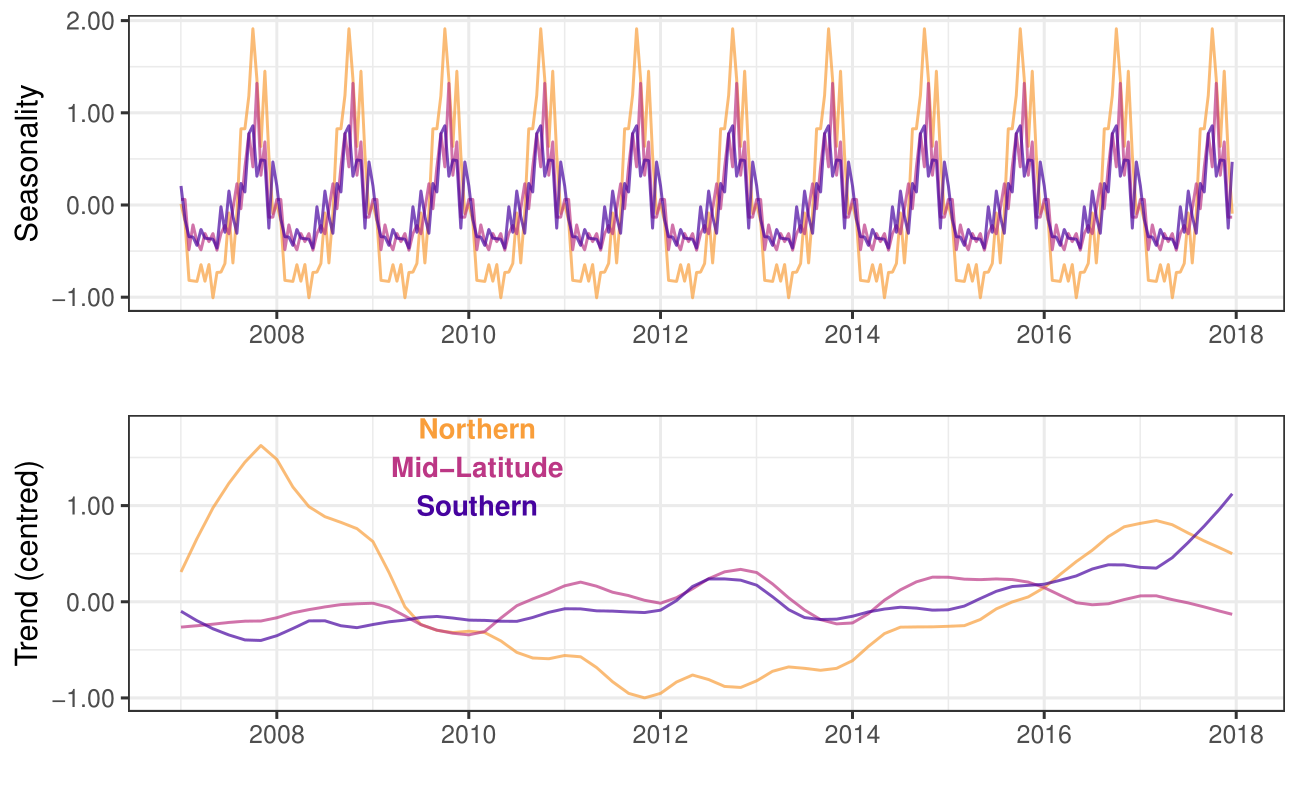

Supplement: S5 Fig — The trend components are centred (xcentre = x–mean(x)) to facilitate simpler comparisons of their temporal dynamics. (TIFF) [file pcbi.1009874.s005.tiff]

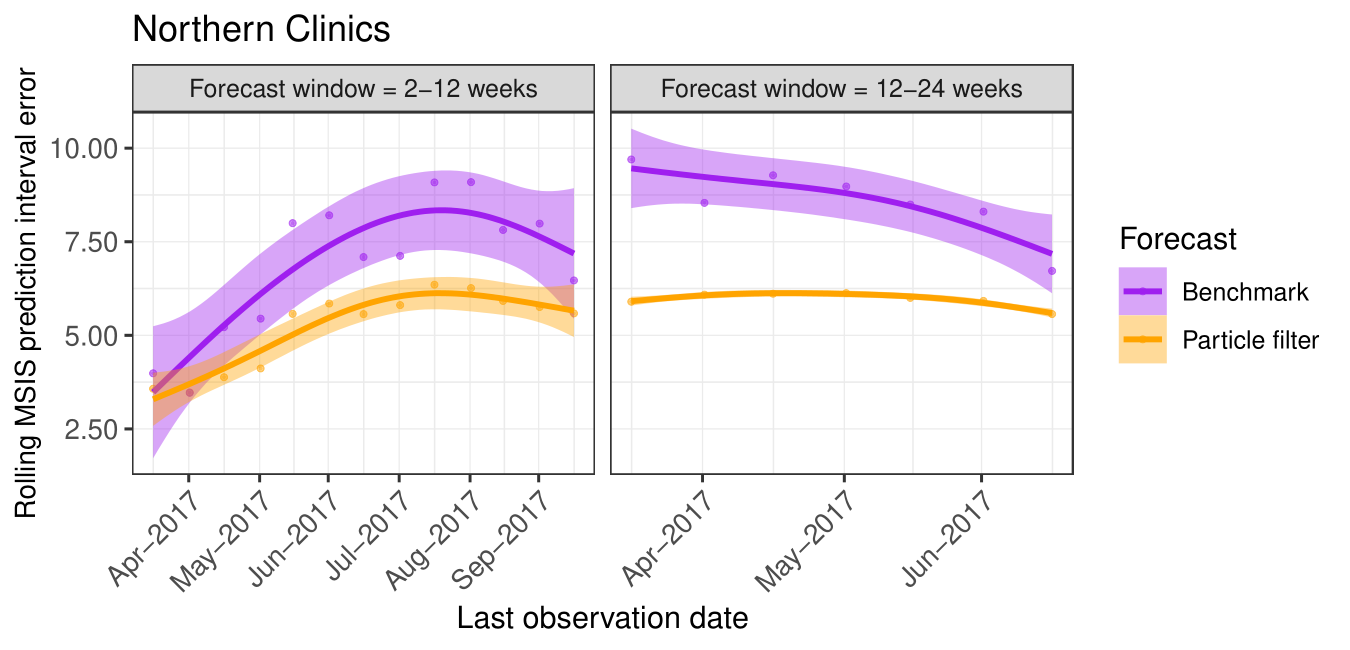

Supplement: S6 Fig — Rolling prediction interval errors for the ETS benchmark (purple shading) and particle filtered ensemble (orange shading) applied to paralysis tick admissions for GreenCross clinics in the northern region. The benchmark model was iteratively retrained on the full dataset as observations became available to simulate a scenario in which models are continually re-calibrated to incoming data. The particle filter involved no retraining for the seasonally adjusted models (ARIMAseasadj and GARCHseasadj), but instead used iterative assimilation of incoming observations via Sequential Monte Carlo. Lines and shaded areas show trends and 99% confidence intervals estimated using cubic regression splines. (TIFF) [file pcbi.1009874.s006.tiff]

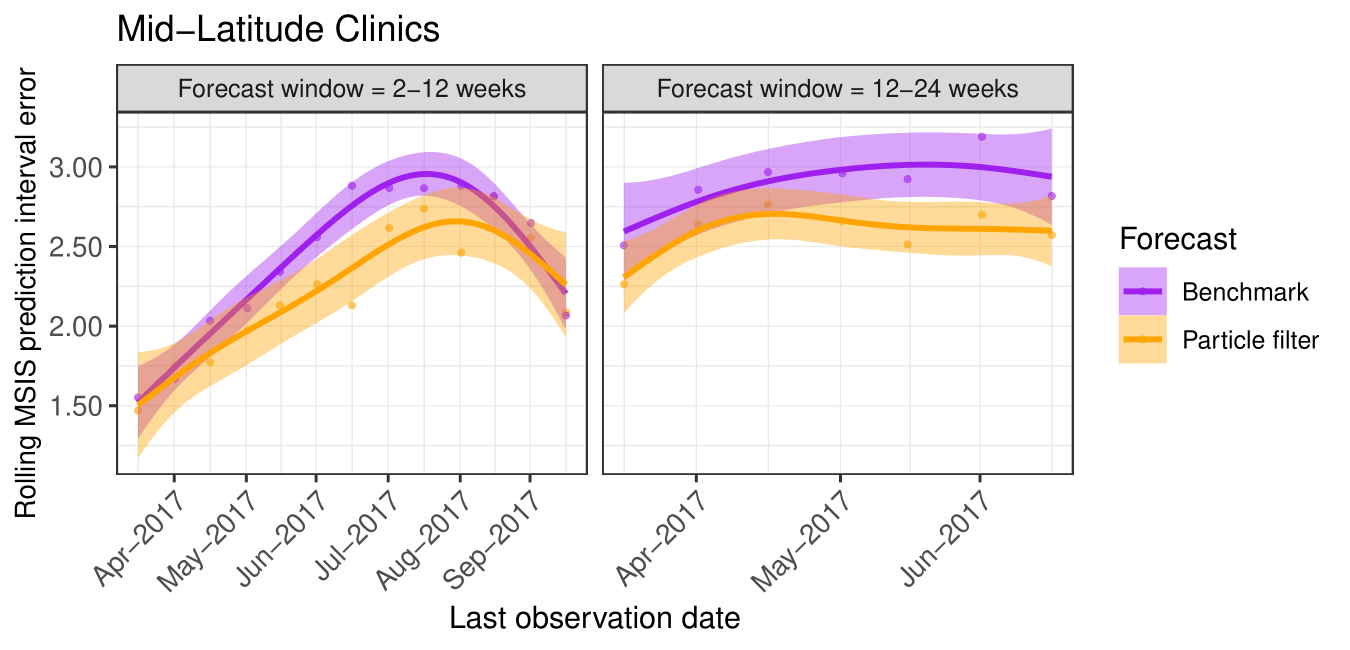

Supplement: S7 Fig — Rolling prediction interval errors for the ETS benchmark (purple shading) and particle filtered ensemble (orange shading) applied to paralysis tick admissions for the mid-latitude GreenCross clinics. The benchmark model was iteratively retrained on the full dataset as observations became available to simulate a scenario in which models are continually re-calibrated to incoming data. The particle filter involved no retraining for the seasonally adjusted (ARIMAseasadj and GARCHseasadj), but instead used iterative assimilation of incoming observations via Sequential Monte Carlo. Lines and shaded areas show trends and 99% confidence intervals estimated using cubic regression splines. (TIFF) [file pcbi.1009874.s007.tiff]

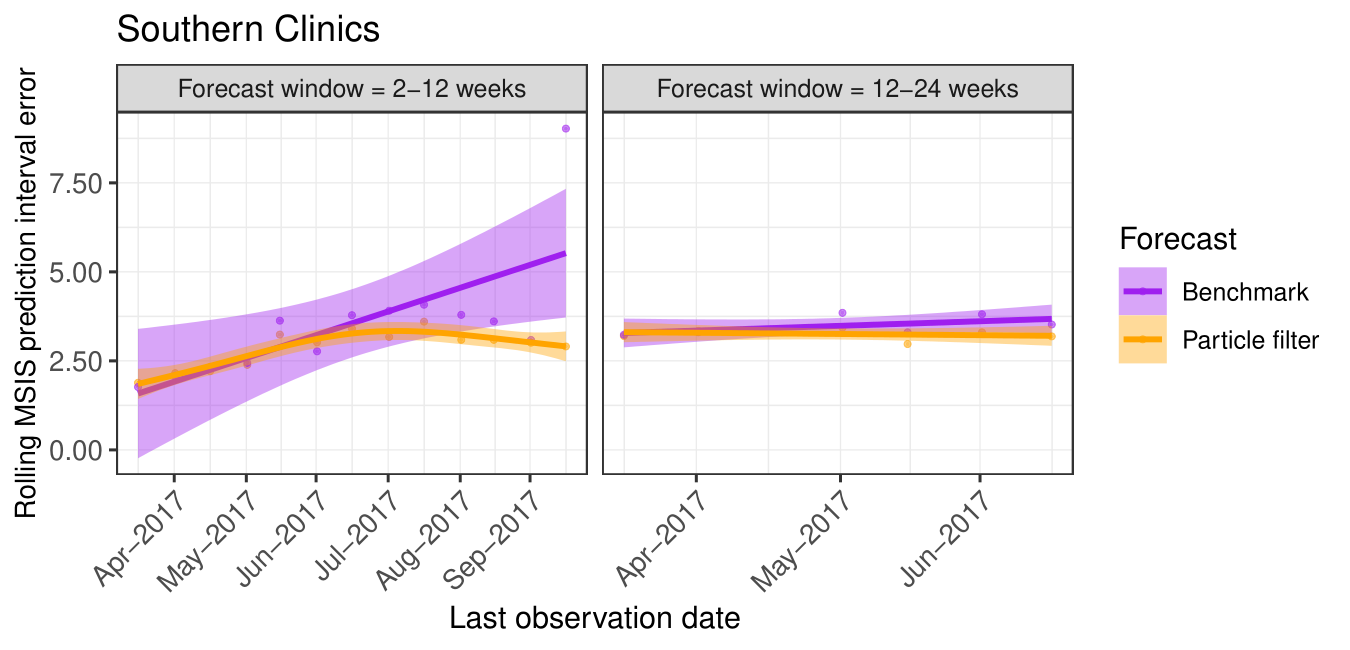

Supplement: S8 Fig — Rolling prediction interval errors for the ETS benchmark (purple shading) and particle filtered ensemble (orange shading) applied to paralysis tick admissions for the GreenCross clinic in the southern region. The benchmark model was iteratively retrained on the full dataset as observations became available to simulate a scenario in which models are continually re-calibrated to incoming data. The particle filter involved no retraining for the seasonally adjusted (ARIMAseasadj and GARCHseasadj), but instead used iterative assimilation of incoming observations via Sequential Monte Carlo. Lines and shaded areas show trends and 99% confidence intervals estimated using cubic regression splines. (TIFF) [file pcbi.1009874.s008.tiff]
