# Supplementary material for: Near-term forecasting of companion animal tick paralysis incidence: An iterative ensemble model
Source: PLoS Comput Biol. 2022 Feb 16;18(2):e1009874. doi: 10.1371/journal.pcbi.1009874 (PMC8887734; doi:10.1371/journal.pcbi.1009874)
Supplement: S1 Table — ARIMA, Autoregressive Integrated Moving Average; GARCH; Generalized Autoregressive Conditional Heteroskedasticity; GAM, Generalized Additive Model; ETS, exponential smoothing. (DOCX) [file pcbi.1009874.s009.docx]

Table S1: Assumptions of predictor models used to forecast paralysis tick admission cases to Sunshine Coast veterinary clinics and build the ensemble forecast. ARIMA, Autoregressive Integrated Moving Average; GARCH; Generalized Autoregressive Conditional Heteroskedasticity; GAM, Generalized Additive Model; ETS, exponential smoothing.

| **Response variable** | **Model** | **Assumptions** |
| --- | --- | --- |
| Seasonal series | ETS_seasonal_ | Response is a weighted moving average of lags with weights that decrease exponentially with time; flexible error, trend, and seasonality |
|  |  |  |
| Seasonally adjusted series | ARIMA_seasadj_ | Response is a linear combination of additive predictor effects and moving average lag effects, with Gaussian error. ARIMA_seasadj_ + ETS_seasonal_ = final ARIMA_seasadj_ forecast |
| Seasonally adjusted series | GARCH_seasadj_ | Response is a linear combination of additive predictor effects and lagged mean effects. Variance at time t is conditional on variances at times t-1 and t-2. GARCH_seasadj_ + ETS_seasonal_ = final GARCH_seasadj_ forecast |
|  |  |  |
| Unadjusted (raw) series | GAM_raw_ | Response is a random draw from a Negative Binomial probability distribution. log(*µ*) is modelled as a linear combination of penalized smooth predictor functions and unpenalized smooth temporal functions |
| Unadjusted (raw) series | Prophet_raw_ | *log(x + 1)* transformed response is modelled using a linear combination of a piecewise linear growth trend, seasonality based on Fourier series and additive predictor effects, with Gaussian error |
| Unadjusted (raw) series | Predictor ensemble | Forecast accuracy is maximized, and error minimized, using weighted combination of ARIMA, Prophet and GAM forecasts |
| Unadjusted (raw) series | ETS_naïve | *log(x + 1)* transformed response is a weighted moving average of lags with weights that decrease exponentially with time; flexible error, trend, and seasonality |
