# Supplementary material for: Near-term forecasting of companion animal tick paralysis incidence: An iterative ensemble model
Source: PLoS Comput Biol. 2022 Feb 16;18(2):e1009874. doi: 10.1371/journal.pcbi.1009874 (PMC8887734; doi:10.1371/journal.pcbi.1009874)
Supplement: Attachment — Submitted filename: Clark_tickforecasting_Reviewer_responses.docx [file pcbi.1009874.s011.docx]

Reviewer #1

Here the authors have built a time series forecasting model using incidence counts of tick paralysis cases. The model is a weighted ensemble of several well known time series models which outperformed a baseline model, providing a proof of concept for a potential forecast for tick awareness to promote public health. This paper is excellent. It is clear, well written, and incorporates all best practices of near-term time series forecasting. Namely that the model uses an ensemble where individual models are weighted by their relative perforgmance, it incorporates new observations via assimilation, compares against a relatively simple base model, and evaluates both point prediction and confidence interval performance. In its current form this manuscript is certainly publishable, and is novel for bringing together the above described aspects of time series forecast and presents everything in supplemental R code.

**Response: We thank the reviewer for the positive feedback**

But I feel it is lacking in that, similar to many forecasting studies, it discusses only the modelling component and not the needed logistics for actually implementing it and making tick forecasts a usable resource. Welch et al 2019 points out the four primary parts of this process, and the current manuscript only addresses one of those. As researchers read this manuscript for the modelling aspect, it would benefit them to be exposed to the bigger picture and how those models fit into and affect a larger system. A “next steps” in the discussion would suffice for this, and would help push the field of ecological forecasting forward beyond just building and evaluating the core models. Some potential points would be:

**Response: Thank you for bringing the Welch paper to our attention. We agree it is important to look beyond a single snapshot study and toward a larger forecasting / data assimilation system. We have carefully incorporated the suggestions below:**

- How feasible is it to get real-time tick counts from clinics to take advantage of the data assimilation? The same goes for the various weather drivers which are part of the model.

**Response: We have added a paragraph in the Discussion to introduce the idea of converting our approach into a dynamic management tool and have moved the section on DiseaseWatchDog into this paragraph for more clarity and justification. Within this section, we discuss how the data we use are advantageous due to their short latency periods: “Several automated ecological forecasting frameworks exist, and we believe that our initial prediction tasks show that tick paralysis admissions are worthy of such a system.”**

**“Welch et al (61) outline four primary stages necessary to implement a dynamic management tool: Acquisition, Prediction, Dissemination and Automation. While we focus primarily on prediction, the data and models we use would facilitate an automated forecast and analysis framework. For example, weather observations provided by LongPaddock (https://www.longpaddock.qld.gov.au/silo/ (29, 30)) are updated nightly (one day latency) with new data from Australia’s Bureau of Meteorology, while the rapidly growing VetCompass Australia database (62) collates clinical data from >300 veterinary clinics around Australia to facilitate near real-time updating of domestic animal health analyses / forecasts” (Lines 623 - 646)**

- Is presenting predicted tick counts a useful way to disseminate this information? Would the public actually change their actions based on that information? This ties directly to the interpretability discussed throughout the manuscript. For example a potentially better way to present the forecasts could be color coded symbols representing discrete bins of current tick activity, similar to color coded bush fire dangers. See the following papers on forecast presentation for more discussions on this, which are largely from the weather community as it’s an understudied aspect in ecology: Morss et al 2008, Gerst et al 2020, Hartmann et al 2002, Raftery 2016

**Response: We agree that application of a dynamic forecast tool requires consideration of stakeholder perceptions, particularly concerning visualization and uncertainty communication. While this is beyond our scope, we now mention this in our Discussion and cite the Raftery study as a useful reference: “The design of a dynamic tick paralysis forecast tool would require inputs from stakeholders on the dissemination aspect, particularly to identify useful ways to visualize forecasts that overcome the common misperceptions about probabilities and uncertainty (63)” (Line 644)**

- If this was implemented and was successful in reducing tick bites, the model would then become ineffective since actual bites, and not risk of tick bites, is the predicted response variable. What could be done then to keep it operational?

**Response: The idea that a forecast can change the future values of an entity is an important point raised by key figures in the forecasting industry (i.e. https://otexts.com/fpp2/what-can-be-forecast.html). We consider it unlikely that our forecasts would have enough of an effect to influence actual admissions. Tick paralysis is a major issue in Australia and local clinicians, together with drug companies, work hard to educate pet owners about preventative medications. Yet we still see prominent admissions, which is likely due to a range of factors including difficulties for pet owners to remember medication schedules. Still, if major reductions did take place we would expect our particle filter to adapt and provide meaningful forecasts**

Some other minor points:

L104: I would not consider three studies a plethora. But you can add Dormann et al 2018, Tebaldi 2007, and Thomas et al 2020 to make it close.

**Response: Thank you for bringing these studies to our attention. We now include them as references**

L158: fitting the ARIMA and GARCH models to the trend + residuals is essentially the same as fitting to the anomaly of the time series, which is appropriate. I would not consider this approach a “state space” model though, since a hidden state is not being estimated at this step (though it is applicable to the data assimilation step). Please remove the state space references (eg. L 366, 388, 470, 739, 757)

**Response: We have** **changed the wording when describing these two models i.e.: “We used particle filtering for the two models that were fitted to the seasonally adjusted series (ARIMA_seasadj_ and GARCH_seasadj_)…” (Line 396)**

**And**

**“The particle filter involved no retraining for the seasonally adjusted (ARIMA_seasadj_ and GARCH_seasadj_)…” Figure captions**

L771: please also deposit the github repository on either zenodo or figshare since github is not a permanent archive.

**Response:**  **We have now deposited the data and code in Figshare (https://doi.org/10.6084/m9.figshare).**

Reviewer #2:

The manuscript presents an interesting methodology for forecasting cases of tick paralysis that is very relevant to the field of vector-borne disease research with useful applications to other vector-borne pathogens. The paper is well written and explains the methodology in detail and is easy to follow.

**Response: We thank the reviewer for the positive feedback**

I have some questions that might help clarify some points.

1. While I appreciate the description of the models in detail, I would like to know the rationale for selecting some of them and not others… for example: why not a random forest model?

**Response: We have added several lines to the text to clarify why we chose these particular models: “The models we implement were chosen because they make different assumptions about time series evolution, can incorporate both seasonality and effects of predictors and because quantification of forecast uncertainty is straightforward. However, our choice of candidate models does not by any means represent an exhaustive set, and we acknowledge that there are many other potentially useful models for analysing timeseries and generating forecast distributions” (Lines 242 - 245)**

2. Why were the temporal lags expressed in weeks when cases were aggregated by fortnights? Shouldn’t they be on the same scale?

**Response: We believe weeks are more intuitive and interpretable, particularly for readers from countries where the term ‘fortnight’ is not commonly used. We have added values for both units to the text so that it is more clear that fortnight was the temporal unit of analysis**

3. Apart from the overall trend, it’s not uncommon to have 2-3 years cycles in tick-borne disease cases driven by the population dynamics of ticks. I guess in a way you can visualize it in the trend, but that also includes the long-term trend… based on the seasonal-trend decomposition based on Loess smoothing could you capture those cycles and separate it from the long term trend (say, driven by ISO)? Could you maybe observe that by analyzing the temporal autocorrelation of the “reminder”? I guess that is what you where adjusting for in the GARCH model?

**Response: We agree this is a useful point to discuss. In our case, the lengths of timeseries were not adequate to investigate possible long-term oscillations, however we have added lines in the Discussion to highlight this as a point of future research: “This may be especially necessary when modelling tick paralysis or tick densities in general, as it is not uncommon for tick-borne disease cases to demonstrate 2-3 years cycles driven by the population dynamics of ticks (63). There are a range of models that can incorporate multiple seasonalities, including the GAM and Prophet models we used here, and future work should investigate whether inclusion of these cycles improves near-term forecasts” (Lines 673 - 676)**

4. What was the spatial resolution of the predictors? I understand the resolution in the original raster files but how were they introduced in the models? Did you use buffers around the clinics? Did you average across the whole study area? Same with the temporal data… did you use the fortnight or the actual date of diagnosis for each case? The only one that is obvious to me is the landcover which was obtained yearly for the whole study area as indicated in the text.

**Response: All spatial data was first aggregated to the SA2 level (i.e. the entire study area) as means of all raster cells from within the SA2 boundary. We have added text to the Methods to clarify this process: “We used an Australian Statistical Area Level 2 shapefile obtained from the Australian Bureau of Statistics (https://www.abs.gov.au/AUSSTATS/abs@.nsf/DetailsPage/1270.0.55.001July%202016?OpenDocument) to extract raster variables for the Sunshine Coast. For all environmental rasters, we first calculated average values for the entire Sunshine Coast study area. We then calculated quarterly (3-month periods: Jan – March, April – June, July – Sep and Oct – Dec) anomalies (referred to herein as variable_Q-anomaly_) as additional predictors to describe how conditions compared to long-term averages for that same period” (Lines 206 - 209).**

**Predictors were aggregated to the fortnight scale for analysis: “All predictors were aggregated as fortnightly means across the Sunshine Coast study area” (Line 219)**

5. When selecting the best model in the ARIMA model, did you have competing models? Did you account in any way for model selection uncertainty?

**Response: We did not account for model uncertainty when selecting the ARIMA, and we did not implement post-estimation correction of significance. We now highlight this point in the Discussion: “We also did not use any post-estimation correction of estimated effect sizes as we were mainly interested in forecastability and less so on whether effects were ‘significant’, but studies designed for inference that wish to focus more on interpretation of estimated effects would need to consider post-estimation adjustments to account for our heuristic model selection strategy” (Lines 664 - 668)**

6. Line 265-266: “This model [GARCH model] was an appropriate alternative to the ARIMA as variations in the reminder series showed alternative periods of volatility.” Did you try an autocorrelation function for the reminder? See point 3.

**Response:**  **We did inspect an ACF function for the seasonally adjusted series and found evidence of substantial autocorrelation up to a lag of two fortnights, justifying the ARIMA. However the GARCH model is useful when the magnitudes of errors exhibit some autocorrelation, which will not be directly captured by the ACF. However we did take steps on the reviewer’s recommendation to justify the use of the GARCH, and have included additional text in the methods: “To detect whether there was autocorrelation of volatility in the seasonally adjusted series, we fit an auto.arima() model and plotted the autocorrelation function of the absolute residuals. This plot showed significant autocorrelation of volatility up to a lag of two fortnights, justifying the use of the GARCH model as an appropriate alternative to the ARIMA_seasadj_” (Lines 290 - 292)**

7. Line 269-271: Could you explain why regularized double exponential priors were used?

**Response:**  **Our prior expectation was that most effects of regressors would be small. These priors regularize effects away from ridiculous coefficient ranges and are appropriate here. We have added clarification in the text: “Rather than selecting among combinations of predictors as above, we included all predictors simultaneously and placed regularized double exponential priors (mean = 0, sd = 0.5) on their coefficients to capture our prior expectation that most effects of regressors on the seasonally-adjusted series will be small” (Lines 294 - 300)**

8. For all models (except for the ARIMA model that is explained): how where the lags selected?

**Response: All models used the same lagged predictors, identified by cross-correlations. This step is explained at: “For each predictor, we selected the lag that showed the strongest cross-correlation with the seasonally adjusted series for inclusion in model testing” (Line 224)**

9. Lines 314-315 (Prophet model): The three predictors were selected by testing in each on its own, why not used the variables for which you have some evidenced based on the previous models? Just again thinking about model selection uncertainty.

**Response: We viewed each model as separate from the others, especially since Prophet operated on the log-transformed raw series while ARIMA modelled the remainder. Our further concern with Prophet was the large number of parameters to be estimated and the lack of options for regularizing priors on regression coefficients. However this was our approach, and other more sequential strategies should be explored to see if they improve forecasts.**

10. I don’t think I completely follow the Prophet model, how does it assimilate new data?

**Response: The Prophet model does not assimilate new observations as it is not a state space model. We added a line to clarify that the Prophet and GAM were simply retrained on all available data when building the particle filtered ensemble: “The remaining models (i.e. GAM_raw_ and Prophet_raw_) were updated as above by retraining on the growing dataset to form the particle filtered ensemble, which we compared to the re-trained ensemble and benchmark models described above” (Line 424)**

11. Table 2 has a few problems: check the signs of the coefficients (for example, in the GARCH model the point estimate is not included in the CI for Maximum temp).

**Response: Thanks for pointing this out. All coefficients have been checked and are now correct.**

12. Line 423: “Three models indicated a significant decrease in admissions with above average maximum temperatures…” But for the GAM model we observe that the coefficient is in the opposite direction compared with the two other mentioned models.

**Response: The GAM F statistic is an indication of how nonlinear or ‘wiggly’ a function is, but it does not indicate directionality of effect. We have added plots of GAM effects in Supplementary files so that readers can visualize the estimated effects. We have also modified the caption: “Note that GAM F statistics only indicate the nonlinearity of the estimated smooth function, not the directionality of the effect” and in the main text to clarify: “Note that GAM_raw_ effects are presented as F statistics that do not reveal the directionality of the effect, only how ‘nonlinear’ it is. Directionality can be interpreted by visualising partial effects plots in Fig S2” (Line 479)**

13. Line 507-509: “This is supported by the Prophet model’s estimate of increased admissions with increasing SOI, etc.” How is the non-linear trend of this model accounting for a effects of extreme rainfall? I would be very careful about interpreting the model results in general… you are not looking at inference but forecasting…

**Response: The previous wording was confusing and misleading. We have removed the line in question and toned down the language this section of the Discussion in general.**

14. Line 523: I do agree that exposure data based on demographic and behavioral data are hard to obtain, but I wouldn’t necessarily call it an stochastic process. It would be interesting to control for admissions in the clinics overall (maybe considering another disease that has no seasonality and it’s not related to tick paralysis).

**Response: This aspect is being investigated in our current work using national data on tick paralysis admissions. More work needs to be done to understand how admissions overall are trending within clinics and whether this information has any power to improve forecasts of tick paralysis admissions**

Minor revisions:

Line 267: ARIMA(0,2) instead of ARMA?

**Response: Fixed**

Line 412-413:When it says October 2008, shouldn’t it be 2009? The peak in Fig 2 seems higher in 2009

**Response: Fixed**

Lines 414-415: Shouldn’t it say 2014 instead of 2014? That would match the Fig 2 trend.

**Response: Fixed**
